# Combating medical misinformation and rebuilding trust in the USA

**DOI:** 10.1016/S2589-7500(24)00197-3

**Published:** 2024-10-07

**Authors:** Clara E Tandar, John C Lin, Fatima Cody Stanford

**Affiliations:** Warren Alpert Medical School, Brown University, Providence, RI, USA; Perelman School of Medicine, University of Pennsylvania, Philadelphia, PA, USA; Weight Center, Massachusetts General Hospital, Boston, MA, USA; Division of Endocrinology–Neuroendocrine, Department of Medicine and Division of Endocrinology–Nutrition, Department of Pediatrics, Obesity Research Center, Medical School, Harvard University, Boston, MA 02114, USA

Mutual trust is the foundation of the physician–patient relationship. However, during the past 50 years, public trust of medical professionals in the USA has declined from 73% in 1966 to 34% in 2012, with trust in physicians and hospitals declining from 71·5% in 2020 to 40·1% in 2024.^1,2^ Factors contributing to this decline could include access to overwhelming amounts of health information, increased social and political divide, and poor health literacy.^3,4^

Medical misinformation further exacerbates this decline in trust. From the beginning of our medical education throughout our careers, physicians are trained to communicate complex scientific and medical information and build trust with patients. As physicians and students actively involved in medical education in the USA, we recommend that medical school curricula comprehensively integrate widespread practical education to teach students how to identify and address medical misinformation.

Although trust in physicians has recently declined in the USA and medical misinformation has proliferated on social media platforms, Americans still cite physicians as their most trusted source of health information.^5,6^ Because of this weakened but still persuasive credibility, medical misinformation created and spread by physicians can be particularly harmful to people who ask physicians for clarification or guidance in understanding new health information.

Years of rigorous medical training might not sufficiently prepare physicians to identify and respond to medical misinformation. Many physicians—inadvertently or intentionally—are major producers and distributors of inaccurate medical information in the USA, spreading medical misinformation on multiple social media platforms.^7^ The physician-authored Great Barrington Declaration potentially bolstered resistance to lockdown policies from both the administration of former President Donald Trump and former members of the European Parliament, and could have contributed to many of the 7 million deaths caused by the COVID-19 pandemic worldwide.^8,9^ Physicians with large numbers of social media followers who disseminate medical misinformation can substantially affect patients as those patients might conflate their social media presence with legitimacy or quality of advice, exacerbating the spread of medical misinformation. A video of a Californian physician questioning quarantine and the efficacy of masks with the caption “some doctors are against the quarantine” has received more than 520 000 views on Facebook alone.^10^

Education about medical misinformation should be integrated into the US medical school curriculum to help future physicians identify it and address its spread. Currently, the Medical School at the University of Minnesota (Minneapolis, MN, USA) and the Pritzker School of Medicine at the University of Chicago (Chicago, IL, USA) have formal courses that medical students can enrol in to learn how to fact-check data, translate scientific literature, and use social media to connect the public with accurate information about the COVID-19 pandemic. Although these courses provide a fundamental background in how to identify medical misinformation and apply communication strategies, they are not accessible or widely available to students at other institutions, are too didactic without a practical component, are taught primarily through online lectures, and do not incorporate one-on-one engagement with patients with varying health literacy regarding questions about medical misinformation. As many US medical schools reduce preclinical classroom time from 2 years to 1–1·5 years,^11^ to provide students with earlier real-world clinical exposure, education about medical misinformation should also be taught early and have a practical emphasis. Medical misinformation education should involve real-world practice in sharing health information and engaging communities to help train students to address misinformation across different cultural and environmental contexts.

Practical education on medical misinformation should also expose students to the same tools that often generate or spread misinformation. Exploration of ChatGPT, social media, and other resources that patients use to answer medical questions could help students understand patient concerns. Schools can train standardised patient actors who ask the same series of questions about medical misinformation obtained through Facebook and other social media platforms. These actors can be trained to present medical misinformation calmly or aggressively, teaching medical students to respond appropriately in various scenarios. Objective structured clinical examinations can be developed to test whether students can empathetically and effectively respond to patient concerns driven by misinformation. These institutional efforts will help create an environment in which students can practice their communication skills and receive constructive feedback without the risk of inadvertently spreading misinformation.

By providing early and varied practical exposure to medical misinformation, we can proactively address physician-spread misinformation, working to rebuild trust between patients and physicians in the USA. With the continued decline in physician–patient trust, exacerbated by pandemics and social media, we need to ensure that US medical practitioners are equipped with the necessary tools to understand and address medical misinformation in both local and online communities.
